# Neglected vitamin K deficiency causing coagulation dysfunction in an older patient with pneumonia: a case report

**DOI:** 10.1186/s12877-022-03327-6

**Published:** 2022-07-30

**Authors:** Qiaoping Wu, Lufeng Wang, Rongqing Zhao

**Affiliations:** Clinical Laboratory of Ningbo Medical Centre Lihuili Hospital, Ningbo University, 1111 Jiangnan Street, Ningbo, Zhejiang Province China

**Keywords:** Vitamin K, Coagulation dysfunction, Cefmetazole, Pneumonia, Case report

## Abstract

**Background:**

The development of coagulation disorders can be dangerous and fatal in the older people, especially those with multiple medical conditions. Vitamin K-dependent coagulation disorders are easily overlooked when anticoagulant drugs are not used and the patient shows no signs of bleeding.

**Case presentation:**

We report a case of a 71-year-old male suffering from pulmonary infection with severe coagulation disorder without bleeding symptoms. He also had a history of Parkinson’s disease, Alzheimer’s disease and cardiac insufficiency. Coagulation tests were normal at the time of admission, prothrombin time (PT) is 13.9 (normal, 9.5–13.1) seconds and the activated partial thromboplastin time (APTT) is 30.2 (normal, 25.1–36.5) seconds. But it turned severely abnormal after 20 days (PT: 136.1 s, APTT: 54.8 s). However, no anticoagulants such as warfarin was used and no bleeding symptoms were observed. Subsequent mixing studies with normal plasma showed a decrease in prothrombin times. Vitamin K deficiency was thought to be the cause of coagulation disorders considering long-term antibiotic therapy, especially cephalosporins, inadequate diet and abnormal liver function. After supplementation with 20 mg of vitamin K, coagulation dysfunction was rescued the next day and serious consequences were effectively prevented.

**Conclusions:**

Overall, timely vitamin K supplementation with antimicrobials that affect vitamin K metabolism requires clinician attention, especially in older patients who are multimorbid, frail or nutritionally compromised, and are admitted to hospital because of an infection that needs antimicrobial therapy are at risk of clotting disorders due to abnormal vitamin K metabolism secondary to altered gut flora, which can exacerbate existing nutritional deficiencies.

## Background

The clotting cascade is initiated by two distinct mechanisms: the extrinsic pathway and the intrinsic pathway. The extrinsic pathway is trggered by tissue factor (TF) released from the damaged tissue. Factor VII (fVII) is activated by binding to TF, then fIX and/or fX is activated by a complex of TF and fVIIa (TF:VIIa). The intrinsic pathway is triggered by fXII when the vessel wall is damaged. Through HK and PK, fXII is activated to fXIIa and afterward fXI is activated to fXIa, following by fIX activated to fIXa and fX activated to fXa. Both pathways converge in the production of fXa. FXa and fV form a prothrombin complex in the presence of calcium ions and phospholipid membranes, which cleaves fibrinogen to fibrin and activates platelets [1].

Vitamin K refers to a group of lipophilic vitamins that are essential for proper hemostasis [2]. Vitamin K dependent coagulation factors include II, VII, IX and X (as well as proteins C, S, and Z). The synthesis of these four coagulation factors and regulatory proteins requires the involvement of vitamin K, which can be synthesized in hepatocyte microsomes. It is obtained mainly from green leafy vegetables in the daily diet and is synthesized by many bacteria residing in the human intestine. Therefore, vitamin K deficiency is rare in healthy adults [3]. Vitamin K deficiency leads to bleeding and causes prolonged PT and APTT. Due to the rapid metabolism of vitamin K, PT can be a sensitive indicator for therapeutic testing. Long-term antibiotic therapy, especially cephalosporins, can cause dysbiosis of the intestinal flora, as well as inadequate diet and abnormal liver function, all of which can lead to vitamin K deficiency and coagulation dysfunction [4, 5].

The older people are susceptible to inflammation caused by infection due to decreased immunity. Coagulation activation thus induced will in turn regulates inflammation [6]. Antibiotics usage is a common way to fight inflammation. Vitamin K-dependent coagulation dysfunction induced by long-term use of antibiotics, especially cephalosporins, is dangerous and urgent for older patients. Therefore, timely vitamin K supplementation requires physician’s attention. In this report, we present a case of vitamin K-dependent coagulation dysfunction in an older patient with pneumonia.

## Case presentation

**Fig. 1:**
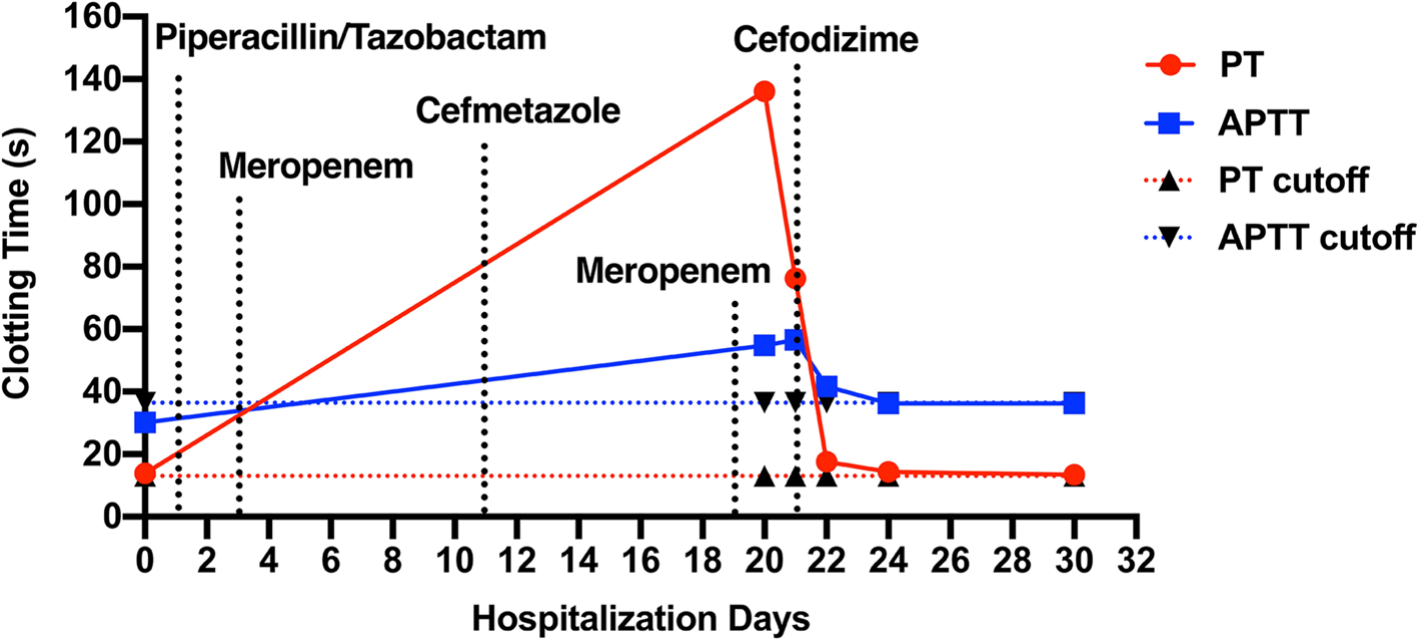
Changes in coagulation functional parameters with time after admission. PT, prothrombin time. APTT, activated partial thromboplastin time

A 71-year-old male patient was admitted to our hospital with pneumonia, and chronic diseases such as Parkinson’s disease, Alzheimer’s disease, lacunar cerebral infarction, and cardiac insufficiency. He had a 15-year history of hypertension and 5-year history of prostatic hyperplasia with stones. The day before admission, the patient’s body temperature rose to 37.6 ℃, with elevated neutrophils and high-sensitivity C-reactive protein (hs-CRP). Physical examination showed crackles in the lungs. Computer tomography (CT) scan showed multiple inflammations in both lungs, suggesting a pulmonary infection. Coagulation tests were normal (PT: 13.9 s, APTT: 30.2 s) (Fig. 1; Table 1). Biochemical tests showed increased urea, 7.29 (normal, 3.60–9.50) mmol/L, B-type brain natriuretic peptide BNP, 1050.0 (normal, 0.0-100.0) ng/L, creatine kinase CK, 172 (normal, 55–170) U/L, its isoenzyme CK-MB, 9.3 (normal, 0.0–24.0) U/L and cardiac troponin I cTnI, 0.575 (normal, 0.000-0.034) ng/ml. Therefore, the patient was treated with piperacillin/tazobactam (4.5 g three times daily, intravenous infusion), as well as symptomatic treatment including reducing phlegm, diuresis and control of Parkinson’s disease symptoms. In addition, Metoprolol and 4100 IU low-molecular-weight heparin were given to control the heart rate and prevent cerebrovascular accidents. Due to dysphagia, a gastric tube was inserted to improve his nutritional status.

**Table 1:** The results of coagulation function examination

Hospitalization days	PT(s)	APTT(s)
0	13.9	30.2
20	136.1	54.8
21	76.2	56.5
22	17.6	41.7
24	14.4	36.2
30	13.4	36.2

*
PT* Prothrombin time, *APTT *Activated partial thromboplastin time

The patient was unable to cough up sputum spontaneously on day 3 after admission and developed a high fever (38.6 ℃), so piperacillin/tazobactam was switched to meropenem (1.0 g three times daily, intravenous infusion) to intensify anti-infective therapy. However, sputum and blood culture results were negative. On day 11 after admission, the patient’s temperature dropped to normal, then meropenem was switched to cefmetazole (2.0 g twice daily, intravenous infusion). On day 15 after admission, the patient again developed fever (38.4 ℃), which was considered to be aspiration pneumonia probably due to prolonged bed rest and neurological disease. On day 18 after admission, the serum potassium value rose to 6.11 (normal 3.50–5.30) mmol/L. Calcium gluconate was used to antagonize potassium toxicity, along with high glucose plus insulin, furosemide, and potassium-free nutrient solution. In view of poor infection control and renal insufficiency, cefmetazole was again switched to meropenem (1.0 g once daily, intravenous infusion) on day 19 after admission.

The patient presented with severe coagulation abnormalities on day 20 after admission (Fig. 1; Table 1). Coagulation tests showed greatly prolonged PT (136.1s) and APTT (54.8s). However, the patient had a normal platelet count, no bleeding symptoms, and had not taken warfarin or other anticoagulant drugs. The results of the mixing study then showed a significantly lower PT (16s) (Fig. 1; Table 1), indicating the lack or anergy of coagulation factors. On day 21 after admission, 20 mg of vitamin K supplementation rescued the coagulation dysfunction (Fig. 1; Table 1). A chest CT showed obvious resorption of the infection lesions of bilateral lungs, so meropenem was replaced with cefodizime (2.0 g twice daily, intravenous infusion) to continue anti-infective therapy. The laboratory results of coagulation function returned to normal on day 22 after admission (Fig. 1; Table 1). In the following days, the pneumonia improved significantly, and the patient was discharged on day 33 after admission.

## Discussion and conclusions

Long-term anti-infective therapy is common in hospitalized patients. Cefoperazone are widely used in China because of its high safety profile and low nephrotoxicity. However, long-term use of cefoperazone may lead to vitamin K deficiency. Several cases of hemorrhage caused by cefoperazone (CPZ) after 5 to 11 days of use have been reported previously [7–12]. Cephalosporins containing side chains of N-methylthiotetrazole (latamoxef, cefmenoxime, cefoperazone, cefotetan, cefamandole, cefametazole) or methyl-thiadiazole (cefazolin) have been reported to be inhibitors of hepatic vitamin K epoxide reductase, leading to a lower nutritional-vitamin K status predisposes to hypothrombinemia, whereas two cephalosporins without a heterocyclic side chain (cefotaxime and cefoxitin) are not [5, 13, 14]. Cefodizime used in this case did not affect hemostasis [15]. It should be noted by physicians that it is necessary to monitor coagulation in older people on long-term antibiotics that affect vitamin K metabolism (Table 2). Long-term antibiotics treatment could kill a large number of normal intestinal flora which influences the synthesis of vitamin K. In addition, Frailty and as such an inadequate diet, abnormal liver function also affects the intake and synthesis of clotting factors and vitamin K. Besides, the diseases that impede fat absorption such as common-duct obstruction and cystic fibrosis, can also lead to vitamin K deficiency. Timely vitamin K supplementation could prevent the serious consequences of hemorrhage.

**Table 2:** Antimicrobials that affect vitamin K metabolism or not

Antimicrobials	Affect vitamin K metabolism (Yes/No)
Latamoxef	Yes
Cefmenoxime	Yes
Cefoperazone	Yes
Cefotetan	Yes
Cefamandole	Yes
Cefametazole	Yes
Cefazolin	Yes
Cefotaxime	No
Cefoxitin	No
Cefodizime	No

Coagulation disorders in the older people are dangerous, so timely prevention of bleeding is essential. In this report, the patient’s coagulation function was normal on laboratory tests at the time of admission. However, after 20 days of anti-infective treatment, the PT value became severely abnormal without any anticoagulant medication. However, the patient did not have any bleeding symptoms. The mixing study showed greatly reduced PT value, close to normal values, suggesting the deficiency or anergy of coagulation factors. Mixing studies are typically used to investigate abnormal clotting time results and help distinguish clotting time prolongation due to a coagulation factor deficiency or an inhibitor. Combined with the patient’s treatment and his condition, vitamin K deficiency was considered to affect the coagulation function, and vitamin K supplementation then rescued coagulation dysfunction and prevented the hemorrhage.

Furthemore, the patients had a 15-year history of hypertension, which may have contributed to the chronic heart failure. Patients with heart failure are more prone to coagulation disorders and are at risk for thromboembolic events, which may derive from many sources, such as blood stasis, low cardiac output, dilated cardiac chambers, decreased activity, increased intracardiac and central venous pressures, rhythm problems, or other accompanying factors such as inflammation, neurohormonal activation, and endothelial dysfunction [16, 17]. However, the interaction of drugs or other potential agents that influence the vitamin K-dependent coagulation function should not be excluded. Overall, our case highlights the importance of the need to monitor coagulation in the older patients on antibiotics that affect vitamin K metabolism, especially for those who are multimorbid, frail or nutritionally compromised.

## Data Availability

Data sharing is not applicable to this article as no datasets were generated or analyzed during the current study.

## Abbreviations

PT: Prothrombin time
APTT: Activated partial thromboplastin time
hs-CRP: High-sensitivity C-reactive protein
CT: Computer tomography
BNP: B-type brain natriuretic peptide
CK: Creatine kinase
cTnI: Cardiac troponin
CPZ: Cefoperazone
